# 2267. Evaluation of Antibiotic Prescribing Trends from a Dental Clinic After Implementation of a Dental Antimicrobial Stewardship Initiative

**DOI:** 10.1093/ofid/ofad500.1889

**Published:** 2023-11-27

**Authors:** Noor M Njeim, Allison Stilwell, Harold Horowitz, Aaron Brandwein, James Sconzo, Claire Droumbakis

**Affiliations:** Memorial Sloan Kettering Cancer Center, Brooklyn, New York; NewYork-Presbyterian Brooklyn Methodist Hospital, Brooklyn, New York; New York Presbyterian-Brooklyn Methodist Hospital, Brooklyn, New York; New York Presbyterian Brooklyn Methodist Hospital, Brooklyn, NY, New York; nyp Brooklyn Methodist Hospital, Brooklyn, New York; New York Presbyterian Brooklyn Methodist Hospital, Brooklyn, NY, New York

## Abstract

**Background:**

Inappropriate antibiotic prescribing by dentists is as high as 80% and in the U.S., dentists rank third for writing the most antibiotic prescriptions. At NewYork-Presbyterian Brooklyn Methodist (NYP-BMH), the Antimicrobial Stewardship Program (ASP) and Division of Dental Medicine collaborated to create a clinical practice guideline regarding antibiotic use in dentistry that was implemented in May 2021 and is reinforced with annual education to dental prescribers. The purpose of this study is to compare outpatient antibiotic prescribing trends pre- and post-guideline implementation.

**Methods:**

Patients presenting to NYP-BMH dental clinic for an emergent indication and prescribed an antibiotic from January-April of 2019-2021 (pre) and January-April of 2022 (post) were included. The primary outcome was appropriateness of antibiotic therapy. Secondary outcomes included a comparison of antibiotic selection, dosing and duration of therapy. A subgroup analysis of patients with reported penicillin allergies was conducted to compare alternative agents prescribed.

**Results:**

A total of 119 patients in the pre and 100 patients in the post-guideline period were included. The primary outcome of appropriate antibiotic prescriptions significantly improved after guideline implementation (pre 45% vs post 64%, p< 0.01). The majority of antibiotic indications were treatment-related and the most common dental diagnosis was caries. The most common antibiotic prescribed in the study was amoxicillin. Compared to pre-guideline, there was significant decreased utilization of penicillin VK (pre 3.4% vs post 0%, p=0.05) and amoxicillin-clavulanate post-guideline (pre 16% vs post 8%, p=0.02). A wide variety of dosing regimens were utilized with more guideline-concordant regimens utilized post-guideline (pre 59% vs post 72%, p=0.04). On average, shorter durations of therapy were prescribed post-guideline (pre 6.8 days vs post 5.7 days, p< 0.02). Penicillin-allergic patients were prescribed less clindamycin after guideline implementation.

**Conclusion:**

ASP initiatives and collaboration with outpatient dental clinics can improve appropriate antibiotic prescribing. As ASPs look to expand into ambulatory care settings, dental clinics should be prioritized.

**Disclosures:**

**All Authors**: No reported disclosures

